# Oral fluid testing can be used to monitor xenotransplant donor herds for porcine cytomegalovirus/roseolovirus status

**DOI:** 10.3389/fvets.2024.1471184

**Published:** 2024-12-19

**Authors:** Susan K. Schommer, Melissa S. Samuel, Kristin M. Whitworth, Addison K. Byrne, Kevin D. Wells, Randall S. Prather

**Affiliations:** ^1^Division of Animal Sciences, National Swine Resource and Research Center, University of Missouri, Columbia, MO, United States; ^2^Division of Animal Sciences, College of Agriculture, Food and Natural Resources, University of Missouri, Columbia, MO, United States

**Keywords:** oral fluid, porcine cytomegalovirus/roseolovirus, real-time PCR, virus safety, xenotransplant

## Abstract

A major concern of xenotransplantation is that donor organs may be a source of pathogens. One pathogen in particular, porcine cytomegalovirus (PCMV), a porcine roseolovirus (PRV), is thought to result in donor organ failure in an immunosuppressed state. Porcine cytomegalovirus is difficult to detect in organ donor swine because of its ability to establish latency. Establishment of an antemortem testing protocol to monitor and maintain PCMV/PRV negative herd status decreases the risk of inadvertently using an organ harboring the virus. Oral fluid has become a common sample for detecting a number of porcine pathogens. A real-time PCR assay was adapted to include an internal control for inhibition and results from antemortem samples (blood, oral fluid) were compared to postmortem spleen from pigs in a known positive herd. When using both oral fluid and blood to test pigs over 12 months of age 13/20 animals with positive spleens tested real-time PCR positive. Animals younger than 12 months of age were tested individually and in group housing with all pigs positive by oral fluid and/or blood. PCMV/PRV testing of oral fluid in young animals and a combination of blood and oral fluid in older animals can be used to verify that a herd has been kept PCMV/PRV free, as in the high biosecurity facility of the National Swine Resource and Research Center.

## 1 Introduction

Porcine cytomegalovirus (PCMV), a porcine roseolovirus (PRV) officially named suid betaherpesvirus 2, is endemic in pigs throughout the world. PCMV/PRV is normally latent in adult pigs but can cause rhinitis, pneumonia, and mortality in pigs < 3 weeks old. Because of its high prevalence, herd immunity to PCMV/PRV generally prevents clinical signs from being observed. Virus shedding begins around 3–6 weeks of age and becomes undetectable at 11–12 weeks; even though the host remains latently infected (1). The advent of real-time PCR methods has extended that detection up to 17 weeks post-infection (2).

PCMV/PRV is a major concern in genetically modified pigs that may be immunocompromised and in animals that will provide organs for xenotransplantation. Human CMV is the most significant post-transplantation infection in human-to-human transplants and baboon CMV has been transmitted to a human via xenotransplant (3). PCMV/PRV has been shown to reactivate when tissues from positive pigs are transplanted to baboons in xenotransplant models. This reactivation significantly decreases the survival time of donor organs (4, 5). During the first heart xenotransplant into a human patient, PCMV/PRV was unknowingly transmitted to the patient despite pre-transplant testing (6). While experimental infection of sows results in transplacental transmission of PCMV/PRV (7, 18), more recent studies that used natural infection determined that virus was undetectable in late-stage fetuses or cesarean-derived piglets, thus acquisition of virus is primarily postnatal (19). By using cesarean-derived pigs and maintaining high-health specific pathogen free herds the likelihood of having PCMV/PRV in xenotransplant donor herds/organs decreases. Such PCMV/PRV-free facilities have also been derived by using an early weaning protocol (8).

PCMV/PRV viral DNA has been detected by using real-time PCR to test blood and different tissues; however viral latency makes detection difficult. Oral fluid, typically collected by having pigs chew on a rope, has become a frequently used sample in animal production medicine over the last 10 to 15 years. Oral fluids are composed of a complex matrix with an assortment of hormones, metabolites, antibodies, and enzymes produced in the mouth as well as elements of the environment that are present due to normal biting, smelling and rooting behaviors (9). Oral fluid collection is consistent with pig's natural behaviors of curiosity and chewing as opposed to other antemortem collections such as swabbing and blood collection that often induce some level of stress. Oral fluids differ from saliva collection by swabs. Oral and nasal swabs are inherently collected from a smaller area and the resulting sample is often diluted to express the sample for processing, which could reduce the target below detection. A wide variety of swine pathogens can be detected in oral fluid via nucleic acid detection as well as antibody detection (10). Detection rates of several pathogens have been found to be greater in oral fluids as compared to buccal or nasal swabs (10). The collection of oral fluids is also less labor intensive and numerous animals can be sampled at one time. Oral fluids are particularly well suited for growing age pigs living in group settings; however, individual and older animals can also be sampled, though the process may be more labor intensive or take more training of the animals (11).

This study measured the feasibility of oral fluids as an antemortem sample for monitoring a PCMV-free herd. Real-time PCR detection of PCMV/PRV was conducted on antemortem blood samples and post-mortem spleen and compared to oral fluid in a known positive herd.

## 2 Materials and methods

### 2.1 DNA extraction and PCR testing

DNA was extracted from blood and oral fluid samples by using the Quick DNA Miniprep Plus kit (Zymo Research) and from spleen by using the NucleoSpin Tissue kit (Clontech). The PCR protocol published by Mueller et al. (12) was modified to use the internal control primers provided with the PCR reagents in the QuantiFast Pathogen + IC kit (Qiagen) as a single tube reaction and optimized by using serial dilutions of gBlock plasmid (IDT) and positive samples (Table 1).

**Table 1:** PCMV/PRV primer, probes and gblock used for real-time PCR.

**Nucleic acid**	**Sequence 5^′^-3^′^**
gblock	GTCAAGGAGA TAGTCAGATT GTTGTTCTGG GATTCCGAGG TTGCCAGGGC GGCGGTCGAG CTCTCTCAGA TGAGCTGCGA CGAAGTGATA CGGAACGGAT TGCCCGCGGG GATACACAAG
Forward primer	GTT CTG GGA TTC CGA GGT TG
Reverse primer	ACT TCG TCG CAG CTC ATC TGA
Probe	6FAM-CAGGGCGGCGGTCGAGCTC-ZEN-BHQ

Briefly, 4 μl of high concentration internal control (Qiagen) was added to 200 μl of the sample prior to extraction. The biological fluids protocol for DNA extraction was followed as written for extraction of the blood and oral fluid samples. For spleen samples, 0.1 g of spleen was finely minced per 1 ml of sterile PBS and crushed with a plastic pestle. From the resulting suspension, 200 μl was transferred to a fresh microfuge tube and centrifuged briefly to pellet the solids. The supernatant was removed and the pellet was resuspended in lysis buffer and proteinase K. Four μl of high concentration internal control was added and incubated in a dry bath at 56°C overnight, completing the extraction as written. Negative controls containing the internal control were included in each extraction.

The PCR reaction mix contained 1 μM of the forward and reverse PCMV polymerase gene primers (12), 0.5 μM probe, and 1X of the master mix components (Buffer, 50X Rox, Internal control primers) and water up to 20 μl per reaction. Extracted sample DNA of 5μl was added for a total reaction volume of 25 μl. PCR was performed at 95°C for 5 min followed by 45 cycles of 95°C for 15 s and 60°C for 30 s (ABI 7500Fast, Applied Biosystems). Positive and negative controls were run with each PCR reaction.

Validation of the assay was performed to determine the slope, intercept and R^2^ coefficient for the assay by using 10-fold dilutions of the gBlock from 10^6^ to 0.1 copies/reaction. Limit of detection and repeatability were conducted across 6 days at 10,000, 100 and 1 copies/reaction with 5 replicates each run resulting in 30 reactions per dilution. The limit of detection in oral fluid and blood was determined by spiking a negative sample with 100, 50 or 20 copies of the gBlock and then extracted resulting in 10, 5, and 2 copies per reaction.

### 2.2 Care of experimental animals and sample collection

Pigs used in this study were clinically healthy domestic and miniature crossbred animals housed at the University of Missouri-Columbia as part of the National Swine Research and Resource Center herd and included both wild-type and genetically modified animals. Fourteen animals used in this study were conventional wild-type pigs that were used as surrogates, the remaining animals were genetically modified or wild-type litter mates. Animals were raised on slatted floors with public district water and sewer. Pigs were housed socially, fed daily, and had unlimited access to water. All procedures performed in studies involving animals were approved and conducted in accordance with the ethical standards of the University of Missouri Institutional Animal Care and Use Committee at the University of Missouri in Columbia, Missouri and compliance with the National Institutes of Health guidelines for the care and use of laboratory animals including the US Public Health Service's Policy on Human Care and Use of Laboratory animals.

Whole blood was collected in EDTA tubes by jugular venipuncture. Oral fluid samples were collected by utilizing all cotton rope cut to ~1 foot in length that had been unwound and held or tied for the pig(s) to chew on. Samples for individual pigs were collected by limiting access to the rope to that pig. When multiple pigs were sampled, the rope fragments were held or hung so that all of the pigs in a pen had access. Oral fluid was expressed from the rope into a 50 ml conical tube by squeezing the length of the rope along the tube rim. Oral fluid and blood samples were collected on the same or successive days and no more than 5 days prior to spleen collection. Pigs for the study were selected from healthy pigs being culled based on herd needs. Pigs were humanely euthanized by intravenous pentobarbital injection according to American Veterinary Medical Association Guidelines on Euthanasia prior to the collection of spleen tissue.

## 3 Results

### 3.1 Characterization of assay performance

The primer and probe utilized in this paper were validated for use with the current protocol which incorporates an exogenous control to detect inhibition of the assay. Initial optimization utilizing a 10-fold dilution of synthesized gBlock resulted in an assay with an R^2^ value of 0.997 and a slope of −3.39 across a dynamic range of 9 logs. Reproducibility across 3 different input quantities resulted in detection even at 1 copy per reaction with a Ct of 33.73 ±0.98 across 30 reactions and 6 independent runs. This resulted in a limit of detection of 1 with 29/30 samples testing positive. To better account for the sample matrix and extraction process, 200 μl oral fluid samples were spiked prior to extraction with the gBlock. At 20 copies per sample PCMV/PRV detected 5/10 times and at 50 copies 9/10 times resulting in a LOD of 5 copies per reaction in oral fluid.

### 3.2 PCMV/PRV antemortem diagnostic testing

For each animal, EDTA whole blood, oral fluid and spleen were collected for testing with the status of the spleen determining if an animal was considered positive or negative. A Ct of ≤ 38 was considered positive regardless of sample type tested. When testing oral fluid 17 of 31 positive animals (55%) were detected (Table 2). If blood was used as the antemortem sample 15 of 31 positive animals (48%) were detected, as well as one animal that had a negative result on spleen (Table 3). However, several animals were only positive in either blood or oral fluid. If an animal was considered positive with a positive result on either sample, 22 of 31 positives (71%) were detected with one additional positive detected on an animal with a negative spleen (Table 4).

**Table 2:** Comparison of PCR on spleen to PCR on oral fluid.

	**PCR on spleen**	
**PCR on oral fluid**	**Positive**	**Negative**
Positive	17	0
Negative	14	6

PCR was performed on individual sample types from animals ages 3 months−2 years old.

**Table 3:** Comparison of PCR on spleen to PCR on whole blood.

	**PCR on spleen**	
**PCR on whole blood**	**Positive**	**Negative**
Positive	15	1
Negative	16	5

PCR was performed on individual sample types from animals ages 3 months−2 years old.

**Table 4:** Comparison of PCR on spleen to PCR on oral fluid and whole blood.

	**PCR on spleen**	
**PCR on oral fluid and blood**	**Positive**	**Negative**
Positive	23	1
Negative	8	5

PCR was performed on individual sample types (spleen, oral fluid, or whole blood). For each animal if the PCR on oral fluid or whole blood or both was positive the sample was positive; animals were negative if both oral fluid and whole blood PCR were negative. This table includes all animals individually tested in this study, ages 3 months−2 years old.

To collect the spleen, euthanasia of the animal was necessary. Because of this many of the animals (26/37) were mature adults over 12 months of age, 14 of which were being culled from the herd after serving as wild type surrogates (Table 5). The remaining 11 animals were under 12 months and 100% (9/9) of those animals were positive by either oral fluid (9/11) or blood (9/11) (Table 6).

**Table 5:** Comparison of PCR on spleen to PCR on oral fluid and whole blood in pigs over 12 months of age.

	**PCR on Spleen**	
**PCR on oral fluid and blood**	**Positive**	**Negative**
Positive	12	1
Negative	8	5

PCR was performed on individual sample types (spleen, oral fluid, or whole blood). For each animal if the PCR on oral fluid or whole blood or both was positive the sample was positive; animals were negative if both oral fluid and whole blood PCR were negative.

**Table 6:** Comparison of PCR on spleen to PCR on oral fluid and whole blood in pigs under 12 months of age.

	**PCR on Spleen**	
**PCR on oral fluid and blood**	**Positive**	**Negative**
Positive	11	0
Negative	0	0

PCR was performed on individual sample types (spleen, oral fluid, or whole blood). For each animal if the PCR on oral fluid or whole blood or both was positive; animals were negative if both oral fluid and whole blood PCR was negative.

Younger animals are also often housed together. Oral fluid samples were collected from young, group housed animals to evaluate the utility of this PCMV/PRV surveillance method. All but 1 of the pens of young pigs (5 months old and younger) had at least 1 animal test positive for PCMV/PRV by using a spleen sample (Table 7). In addition, every pen was positive for PCMV/PRV by real-time PCR on a pooled oral fluid sample.

**Table 7:** Comparison of PCR on spleen and blood from individual pigs to PCR on oral fluid from the pen.

**Litter #**	**Age**	**Spleen**	**Blood**	**Oral Fluid (sample per pen)**
128	6 weeks	2/5	0/5	1/1
126	7 weeks	2/2	2/2	1/1
116	8.5 weeks	1/6	0/6	2/2
81	4 months	2/2	2/2	1/1
P96/P97	5 months	2/2	2/2	1/1

## 4 Discussion

PCMV/PRV is of concern in animals whose organs will be used for xenotransplantation, as illustrated by the fact that this virus was transmitted to the first human patient to receive a pig heart (6). This virus is also a concern in genetically modified pigs that may have mutations making them immunocompromised. Breeding colonies used for xenotransplantation may have as many as 95% of the animals PCMV/PRV positive (12). In order to address these concerns, two primary methods of producing PCMV/PRV free animals have been utilized, cesarean delivery (19) and early weaning (8) in the creation of closed high health breeding herds specifically for xenotransplantation. Due to latency of the virus in adults, a careful testing program must be administered to ensure the facilities stay clean of PCMV/PRV as well as other viruses. Inadvertent introduction of PCMV/PRV can go undetected and result in reduced survival rates of transplanted organs (4). While maintaining specific pathogen-free pigs takes dedicated facilities and effort, testing for elimination of PCMV is not feasible as there is limited accessibility to validated serological assays and detection of the virus even by highly sensitive PCR assays is problematic due to the uneven distribution of the virus and its ability to establish latency (13).

Numerous testing strategies have been suggested over the past few years (2). Our study sought to find an antemortem testing scheme that would be able to detect if there was a breach of PCMV/PRV into the system, such as the one that was retrospectively discovered by Mueller et al. (12). Even a closed high health breeding herd will have new introductions to populate with new genetic modifications. Both cesarean-derived hand raised piglets, and an early weaning protocol have been shown to successfully produce PCMV/PRV-free pigs (8, 19) but there is still a risk of introduction at any time via animal, fomite, or personnel movement.

Clark et al. previously examined PCMV/PRV distribution in a variety of tissues and antemortem samples in both adult pigs and piglets aged 19 to 34 days. Spleen was the tissue consistently positive in both groups and why it was used as the standard for comparison in this study. The adult pigs in Clark's study were all negative on antemortem samples including urine, feces, serum, peripheral blood mononuclear cells (PBMC), nasal swabs and saliva swabs while the young pigs had some positives in serum, PBMC and swabs (19). In a later paper young, 10-day old, piglets were tested for PCMV/PRV by using antemortem samples that included serum, anal swabs, oral swabs and ear biopsies (14). By using a duplex real-time PCR similar to the one in this paper, these young piglets were all determined to be positive by oral swab and a few were positive by ear biopsy, while some serum and anal swab samples were weakly positive when the duplex was separated out into uniplex assays. The authors noted that these were all suckling piglets and that the milk itself was not tested and may have contributed to the number of positives.

Oral fluid is a more complex sample than a nasal or oral swab and can be collected in a passive and less stressful manner than swabs or drawing blood. PCMV/PRV is characterized as causing rhinitis and conjunctivitis with the primary sites of virus replication believed to be the nasal mucous glands, the lachrymal glands, or the Harderian glands (15) and oral fluid has the potential to include those secretions. Previous studies found blood samples to be negative for PCMV/PRV especially in older pigs. In our study 15/31 pigs with a positive spleen were also positive by blood, including 7 animals that were more than 12 months old. Oral fluid had a similar level of sensitivity at 17/31 samples PCR positive for PCMV/PRV. Since some animals were positive by only 1 test or the other, the overall detection rate was raised to 71% if either test was positive. There was one animal that was positive on blood but negative on spleen. Both sample types were tested multiple times, with the same result. While spleen is generally the most consistently positive tissue sample some studies have demonstrated that an animal can be positive in several samples while having a negative test in samples from the spleen (15).

As PCMV/PRV becomes latent after the initial infection, detection in antemortem samples is expected to decrease with age. When using both oral fluid and blood to test pigs over 12 months of age 12/20 were positive from animals with positive spleens, which was higher than expected. About half the adult animals (14) were wild-type pigs that had undergone embryo transfer, which may have increased their physiological stress and could contribute to the virus being reactivated and more detectable in antemortem samples than previous studies. However, four embryo transfer animals were also among those with a PCR positive spleen and PCR negative oral fluid and blood. Additionally of the 6 animals with negative spleens, three were surrogate animals and three were from the known negative herd. Adult males were also equally distributed between positive and negative PCR results for circulating virus (3 positive, 2 negative). A recent paper looking at adult wild boars over 22 months of age in Italy and Germany found circulating PCMV/PRV by PCR detection in 44% and 60% of animals respectively, which is consistent with our 50% positive rate in adults (16). The causes of reactivation of the virus are not well understood. The research source herd contains a broader range of ages within each facility than a conventional farm herd, with new animals entering the facility on a regular basis. This may increase exposure to circulating virus from young animals, which when coupled with genetic modifications may contribute to the high level of detection in adult animals.

For the pigs under 12 months of age, 9/11 were positive by blood and 9/11 were positive by oral fluid with all of them positive by one sample or the other. We then further investigated the use of oral fluids as a pen-based sample rather than individual on younger pigs as this is how they are typically housed. With ages ranging from 6 weeks to 5 months, all pens were positive by oral fluid while 3 were negative by blood. The use of pen-based oral fluid sampling increases the ease and number of animals represented in surveillance testing.

Serology is a useful surveillance tool, as it is not dependent on detecting the virus during its replication phase. There are not any validated commercially available serologic assays or reagents for PCMV/PRV. Plotzki et al. did describe a Western Blot assay that can detect specific antibodies to PCMV/PRV (17). This assay has been proposed as part of the screening process for xenotransplant donor pigs after the inability to detect virus until after surgery in the first human heart xenotransplant (2). This panel of testing is critical for individual animals being considered as donors. However, herd monitoring relies on testing a larger number of animals on a consistent basis which is hampered by the lack of reagents.

Continually monitoring a Specific Pathogen Free herd is a critical part of maintaining the status. In the bio secure National Swine Resource and Research Center, PCMV/PRV assays as described here are performed on oral fluid as part of our quarterly surveillance with no positives detected. Samples from the herd are also tested independently as part of xenotransplant protocols further confirming these results. Oral fluid samples can be used to test a pen of animals in a single sample, increasing the power of routine antemortem testing when performed on pigs younger than 12 months of age. PCMV/PRV testing of oral fluid in young animals and a combination of blood and oral fluid in older animals can be used to verify that a herd has been kept PCMV/PRV free. This testingcoupled with the testing of tissues, especially spleen, from culled and sick animals establishes that the herd has remained negative, decreasing the risk of inadvertent transmission when animals are used for xenotransplantation and increasing the number of potential donors.

## Data Availability

The raw data supporting the conclusions of this article will be made available by the authors, without undue reservation.
